# Dyspepsia

**Published:** 1871-07

**Authors:** 


### DYSPEPSIA.

Doctors, like preachers, intend that you shall do as they *say* not as they *do*. We would like to take a considerable portion of this advice to ourself, for we are as much in need of it as any one with whom we are as well acquainted; but, circumstances conspire to keep us at home, and cause us to work more for the benefit of others than for our own well-being. Now, we can give you good, sound advice, and that which will certainly cure your dyspepsia, if you will only be more obedient than we are, and heed it. Dyspepsia is one of the most provoking of maladies—a man is too sick to live, yet not sick enough to die, and often he would willing flip a copper to see which it should be, if that process would only effectually decide the matter.

We have a friend who has dyspepsia—has it "bad," as the boy had the itch; we will tell you how he is afflicted, for his symptoms are about as distressing as any we are familiar with. He is constantly annoyed by hunger—one that food will not appease, because he cannot eat. He spends from eight to twelve hours in his counting room, figuring and managing his extensive business, then retires to his home to consult his works on cookery, to see what the deuce he can eat. He tries oat-meal porridge—it distresses him; cracked wheat and milk is worse; brown bread, beef tea, milk punch, everything that his ingenuity or receipt book can suggest, all aggravate his symptoms. His mind is constantly on his malady, save when he is in his counting room, absorbed in business, hence is most of his time spent there, until exhausted nature lays him upon his back, in bed, where he is allowed to moralize upon his condition all alone, for he is so crabbed that no one else can approach him. If there is any species of humanity more cross and morose than a dyspeptic, we have yet to meet with it, because "we know how it is ourself." Well, our friend devotes all his energies to concocting something to put into his miserable stomach that will remain there without producing pain. In the meantime, his stomach is becoming so irritable, that everything that is taken into it becomes decomposed and putrid, from want of proper digestion, is expelled into the bowels where it irritates and inflames every part with which it comes in contact, giving rise to diarrhœa and hæmorrhoides. Then our dyspeptic's misery becomes complete—he's a complete encyclopedia of all the pains and aches ever experienced by mortal man, and in a frame of mind not calculated to philosophize, with any degree of accuracy, over what is the best plan for him to pursue. We will therefore step in and advise him: Quit your store—go abroad, —a sea voyage to some distant country would be best. If this cannot be afforded, take your rod and gun, and go into the woods, and remain there for a month or two, or, until such time as your brain is as much rested as your stomach. Eat anything you can get your hands upon, except your powder and shot; keep that to secure your food with. Build you a slab shanty, or pitch your tent by the side of some stream where you can catch sufficient trout to keep your table supplied—the more scarce the trout, the better, as you will then have to walk farther and work harder to get a sufficient supply to appease your appetite. Take your oat meal, graham bread, and black tea with you, rely upon your rod and gun to bring the rest of your food. Take a companion with you, and have a real jolly, good time; if you don't return home, in less than two months, cured of dyspepsia, you are really in a very bad condition, and the most sensible thing you can do is to give up all business entirely, and devote the remaining time you have to live to making yourself as comfortable as possible. But don't try to cure your dyspepsia by eternally dieting, while working your brain tired in your store. It can't be done, and the sooner you abandon that experiment the sooner you can place yourself in position to effectually cure your dyspepsia—by rest from all kinds of business, life in the open air, and the diet of a backwoodsman.

